# Interpersonal Values and Academic Performance Related to Delinquent Behaviors

**DOI:** 10.3389/fpsyg.2016.01480

**Published:** 2016-10-17

**Authors:** María Del Mar Molero Jurado, María Del Carmen Pérez Fuentes, Antonio Luque De La Rosa, África Martos Martínez, Ana Belén Barragán Martín, María del Mar Simón Márquez

**Affiliations:** Department of Psychology, University of AlmeríaAlmeria, Spain

**Keywords:** interpersonal values, academic performance, delinquent behaviors, secondary education, adolescence

## Abstract

The present study analyzes the relation between delinquent behaviors, interpersonal values, and academic performance. It also analyzes the possible protective function of interpersonal values against delinquent behaviors. The Interpersonal Values Questionnaire (IVQ) was used to assess interpersonal values, and the Antisocial-Delinquent Behaviors Questionnaire (A-D) was employed to assess antisocial behaviors. The sample was made up of 885 students of Compulsory Secondary Education, aged from 14 to 17 years. The results show that individuals who fail a subject as well as those who repeat a course present higher means in delinquent behaviors. Repeaters present higher means in the values of recognition and leadership, and non-repeaters in the value stimulation, whereas students who do not fail obtain higher scores in the value benevolence. Students with high levels of recognition, independence, and leadership, as well as students with low levels of conformity and benevolence display significantly higher levels of delinquent behaviors. Lastly, the probability of presenting a high level of delinquent behaviors is greater in individuals with: high independence, high leadership, high recognition, low benevolence, and low conformity.

## Introduction

The presence of behavior problems during childhood and adolescence is currently a phenomenon causing great concern (Thomas, 2010). These problematic behaviors frequently lead to antisocial and/or delinquent behaviors with negative consequences for the development and psychosocial adjustment of the adolescent (Fuentes et al., 2011; de la Torre et al., 2013; Gázquez et al., 2015a). In spite of the fact that delinquent behavior includes a large variability of manifestations (Martínez and Gras, 2007), course, and prognosis (White and Frick, 2010), there is a consensus among authors about a series of common traits: these behaviors are a threat to the integrity of others, they infringe social and juridical norms, they are notably frequent and intense, and they are a risk for development and they interfere especially in the individual's processes of adaptation (Garaigordobil, 2005; Peña and Graña, 2006; Burt and Donnellan, 2009; Pahlavan and Andreu, 2009). Thus, taking into account the complexity of the construct, we could refer to a continuum that begins with problem behaviors, passing through antisocial behavior, and ending with delinquent behaviors, of greater severity and social scope.

One of the topics that has received the most attention in the study of delinquent behavior is the analysis of the factors that intervene in the origin and maintenance of this type of attitudinal/behavioral repertories. The more traditional hypotheses point toward certain personal variables as the main triggers of delinquent behavior. In the same vein are the notable contributions like that of Patrick et al. (2009), which refer to two personality dimensions (Impulsivity/Emotional insensitivity) that could be directly related to the presence of severe behavior problems and participation in delinquent actions (Lynam et al., 2009). The presence of psychopathic personality traits has also been indicated as one of the triggering factors of severe patterns of antisocial/delinquent behavior in children and adolescents (López-Romero et al., 2011). In other cases, sensation seeking is proposed as one of the characteristic traits of adolescent personality that, along with the lack of control of impulses, favors the subject's involvement in risk behaviors (Peach and Gaultney, 2013; Pérez-Fuentes et al., 2015). According to Harden et al. (2012), this adolescent tendency to seek sensations is mainly due to changes in personality, explained by genetic factors. Thus, changes in sensation seeking would partially explain a greater proclivity to delinquency during adolescence.

In spite of studies that separately analyze the factors involved in the origin of delinquent behavior, the current tendency is based on a multidimensional and dynamic approach, in which the proposed variables must be considered as part of a compendium and in continuous interaction (Muñoz and Navas, 2004). Thus, we found works analyzing the relation between emotional intelligence and aggressiveness (Inglés et al., 2014), behavior problems (Siu, 2009), and antisocial and delinquent behaviors (Garaigordobil and Oñederra, 2010).

On the other hand, authors like Van der Graaff et al. (2012) point to the moderating role of empathy in the perception of parents' support and their children's performing delinquent actions. These authors found that adolescents with lower empathy had a more negative perception of the support received from their parents and they presented a greater number of delinquent behaviors.

Parenting styles and the characteristics of family relations may be the elements that have received the most attention in the analysis of problem behaviors, either as risk or protection factors (Martínez et al., 2013). In any case, the efficacy of the interventions reveals the importance of family factors as a cause of and/or solution to this problem (Tolan et al., 2013). Concerning the family context, report that children's exposure to episodes of domestic violence and frequent conflicts between the parents is related to the onset of aggressive and delinquent behavior in adolescence. Likewise, other noteworthy works on attachment and delinquent behavior (Sousa et al., 2011) indicate that the establishment of stronger ties with the parents predicts a lower risk of delinquent behavior in adolescence, regardless of the degree of exposure to violent episodes during childhood. In other cases, interest is drawn to the study of the effects of parental control (Harris-McKoy and Cui, 2013) and the use of discipline (Lansford et al., 2011), as key aspects in the origin and maintenance of delinquent behaviors in adolescents.

In the school setting, in addition to the presence of delinquent behaviors, the increasing frequency of academic failure is another concern (Pérez-Fuentes et al., 2011). Many authors (Briggs-Gowan and Carter, 2008; Gázquez and Pérez-Fuentes, 2010; Preddy and Fite, 2012), coincide in relating involvement in delinquent actions to low academic performance, leading to failure, and school dropout (Henry et al., 2012). In this regard, the self-concept as a correlation of social adaptation in adolescence (Fuentes et al., 2011; Álvarez et al., 2015) plays an essential role. Jiménez et al. (2007) found that a positive academic self-concept fulfills a protector function against the development of delinquent behaviors. Likewise, authors like Nakamoto and Schwartz (2010) state that involvement in violent episodes will have a negative effect on academic performance. Ma et al. (2009) note that aggressors perceive their competences as being more impaired and, therefore, they obtain worse academic results. On the other hand, the expectations of self-efficacy and the academic goals give rise motivational profiles (Valle et al., 2015) and may be detrimental to academic performance, in the cases involving aggressors.

Problems relating to the peer group can derive in academic difficulties, the development of violent interactions in childhood, or the amplification of behavior problems in adolescence (Dishion and Tipsord, 2011). At this point, especially during adolescence, the processes of peer influence determine psychosocial adjustment and the acquisition of certain interpersonal values that will guide relations with the peer group (Paciello et al., 2013; Gázquez et al., 2015b). According to Knecht et al. (2010), adolescents select other group members as friends as a function of the level of similarity in interpersonal values. Therefore, processes of influence and adaptation of antisocial/delinquent behavior among its members will take place in the peer group.

In addition, both in the family context and in the peer group, the acquisition of certain interpersonal values—positively or negatively related to delinquent behavior—is implied, an aspect that the present study attempts to examine. Recently, others proposals show the predictive value of social support in the emotional intelligence of adolescents (Azpiazu et al., 2015).

On the other hand, the time dedicated by adolescents to the use of internet and inappropriate videogames has been related to the acquisition and change in the values of youth, and may be associated with a higher probability of delinquent activities (Holtz and Appel, 2011). This is why more attention has been paid in recent years to the variables that make the onset of antisocial behaviors less likely (Inglés et al., 2015) or that attenuate their manifestations after they have emerged (Loeber and Farrington, 2012). Thus, attitudes and values, such as social sensitivity, prosocial leadership, or safety in interpersonal relations, have been related to competence and adequate social adaptation in adolescents (Jiménez and López-Zafra, 2011).

Lastly, in order to provide greater clarity in this regard, we present this work, which will attempt, on the one hand, to analyze the influence of academic performance (measured as failing a subject or repeating a course) on delinquent behaviors and interpersonal values (Hypothesis_1_ = Poor academic performance is associated with a greater presence of delinquent behavior). On the other hand, it also analyzes the relation between high or low scores in interpersonal values and the presence of delinquent behaviors in secondary education students (Hypothesis_2_ = Subjects with high levels of recognition, independence and leadership, and low levels of conformity and benevolence, have higher levels of delinquent behavior). Lastly, we wish to analyze the degree to which interpersonal values fulfill a protective function against delinquent behaviors in secondary education, as well as the interaction of academic performance with interpersonal values and its impact on the presence of delinquent behaviors (Hypothesis_3_ = The presence of high levels in some interpersonal values such as benevolence, exert a protective function against delinquent behavior, with a mediator effect of academic performance).

## Materials and methods

### Participants

The initial sample was made up of 1055 students from the 3rd and 4th grade of Compulsory Secondary Education (CSE) of Almeria province (Spain), of whom 120 (11.37%) were eliminated because they were aliens and had not completed the questionnaires in time due to their lack of mastery of the Spanish language; additionally, due to errors or omissions, or to not having attended one of the two administration sessions, another 50 (4.74%) subjects were excluded. The final sample was made up of 885 students of CSE, of whom 49.8% (*n* = 441) were male and 50.2% (*n* = 444) were female, with age ranging from 14 to 18 years, mean age of 15.2 years (*SD* = 0.90) for the total sample, and 15.22 years (*SD* = 0.92) and 15.19 years (*SD* = 0.89) for males and females, respectively.

The distribution of the sample as a function of having failed a subject was as follows: those who failed a subject (*n* = 729; 377 males and 352 females) and those who did not fail (*n* = 156; 64 males and 92 females). The chi-square test of homogeneity of the frequency distribution, ${\text{c}}_{\left(1885\right)}^{2}$ = 5.87, *p* = 0.02, revealed statistical differences between the four groups made up of the variables Gender and Failing. However, regarding the variable repeating a course: Repeaters (*n* = 273; 139 males and 134 females) and Non-repeaters (*n* = 612; 302 males and 310 females). In this case, no statistical differences were observed among the four groups made up of the variables Gender and Repeating, ${\text{c}}_{\left(1885\right)}^{2}$ = 0.19, *p* = 0.67.

To obtain the sample, we used random cluster sampling, attending to the different geographical areas of the province of Almeria (center, east, and west). Each area had at least one public school, with the sample of each area always exceeding 200 students [center 212 subjects (24%), east 333 subjects (37.6%), and west 340 subjects (38.4%)], four classes in each school (two classes of 3rd grade and two of 4th grade).

### Instruments

#### Academic performance

This was measured with the items: *Did you ever fail a subject? Have you ever repeated a course?* In both cases, the response options were YES/NO.

#### Interpersonal values questionnaire (Gordon, 1977)

This 90-item instrument has two response options (YES-NO) and analyzes six aspects of the individual's relationship with others: Stimulation, Conformity, Recognition, Independence, Benevolence, and Leadership.

#### Antisocial-delinquent behaviors-questionnaire (Seisdedos, 1995)

This includes a total of 40 items that assess antisocial (entering a forbidden place, throwing rubbish on the floor) and delinquent behaviors (using drugs, stealing, etc.).

Internal consistency was analyzed by the coefficient Kuder-Richardson (KR-20) for each of the scales of Interpersonal Values Questionnaire (K-R20_S_ = 0.74; K-R20_C_ = 0.81; K-R20_R_ = 0.77; K-R20_I_ = 0.81; K-R20_B_ = 0.85; K-R20_L_ = 0.78), and scale of criminal behavior of Antisocial-Delinquent Behaviors Questionnaire (K-R20_ADd_ = 0.87). In general, the internal consistency coefficients obtained for scales in the study sample were high (>0.70), indicating adequate homogeneity among the items of the questionnaires.

### Procedure

We contacted the headmasters and guidance counselors of the selected schools to present the goals of the study and the instruments to be used therein. If they expressed interest in participating, we requested their permission and the necessary collaboration to carry out the study. This study was exempt from ethical approval, because the study did not involve any potential risk for the participants. All participants provided written consent. We held a meeting with the parents and the principal researchers and, after informing the parents, we obtained their consent for their children to participate in the study. We then scheduled the application of the questionnaires. The questionnaires were administered in two 50-min sessions, with a variable resting time between them, separated either by a class and a recess, or simply a recess, with more than 20 min between sessions. The questionnaires were administered collectively in the classroom or in one of the spaces of the school if various classes were grouped together. The questionnaires were voluntary and anonymous.

### Data analysis

For the present study, we used a cross-sectional, descriptive, and correlational design in order to determine the relations between interpersonal values (stimulation, conformity, recognition, independence, benevolence, and leadership) and delinquent behaviors, as well as the relationship between these two aspects with the subjects' academic performance, measured as a function of having failed a subject and having repeated a course (failing and repeating).

After the normal distribution of all the SIV scales (Gordon, 1977) had been determined, we identified the criterion to define the thresholds (high and low) of the sample on these scales. Thus, the total sample of subjects (*N* = 885) was divided into two groups for each one of the scales: (a) students with low scores on Stimulation, Conformity, Recognition, Independence, Benevolence, and Leadership, that is, who obtained scores equal to or lower than percentile 25 (scores equal to or higher than 14, 11, 8, 13, 14, and 7, respectively; *n*_2S_ = 233; 26.3%; *n*_2C_ = 235; 26.6%; *n*_2A_ = 218; 24.6%; *n*_2I_ = 237; 26.8%; *n*_2B_ = 262; 29.6%; *n*_2L_ = 240; 27.1%); (b) students with high scores on Stimulation, Conformity, Recognition, Independence, Benevolence, and Leadership, that is, who obtained scores equal to or higher than percentile 75 (scores equal to or higher than 20, 19, 15, 21, 22, and 14, respectively) (*n*_1S_ = 291; 32.9%; *n*_1C_ = 227; 25.6%; *n*_1A_ = 238; 26.9%; *n*_1I_ = 268; 30.3%; *n*_1B_ = 248; 28%; *n*_1L_ = 246; 27.8%).

We used Student's *t*-test to analyze the differences between individuals with high and low scores on the SIV Questionnaire scales, between students who had/had not failed, as well as between students who had/had not repeated a course, regarding delinquent behavior. To determine the magnitude of the effect size of the significant differences yielded by the *t*-test, we used Cohen's *d* index, the interpretation of which is: *d* ≤ 0.50 indicates a small effect size; *d* ≤ 0.79 indicates a medium effect size; and *d* ≥ 0.80 indicates a large effect size.

In order to analyze the predictive capacity of interpersonal values and academic performance on delinquent behaviors, we performed binary logistic regression analysis, using the forward stepwise regression procedure based on Wald's statistic. Thus, the six predictor variables (stimulation, conformity, recognition, independence, benevolence, and leadership) and the criterion variable (delinquent behavior) were divided as a function of high and low thresholds, maintaining for the predictor variables the one used in the previous test. Regarding the predictor variables failing and repeating, it was not necessary to establish any threshold because the students were grouped as a function of whether or not they had that characteristic. To classify the sample according to delinquent behavior, we followed the same criterion as with the SIV Questionnaire, dividing the sample into subjects with high and low scores as follows: (a) subjects scoring high in delinquent behavior were those who scored equal to or higher than percentile 75 (scores equal to or higher than 3; *N*_1_ = 247; 27.9%); (b) subjects with low scores in delinquent behavior were those who scored equal to or lower than percentile 25 (scores equal to 0; *N*_2_ = 373; 42.1%).

This model allows determining the probability of occurrence of a certain fact or event (e.g., aggressive behavior) in the presence one or various predictors (e.g., high scores in stimulation, conformity, recognition, independence, benevolence, and leadership, failing, or repeating) using the Odds Ratio (OR) statistic to estimate this probability both in the total sample and in the sample as a function of the variables gender, failing, and repeating.

Lastly, to analyze conjointly the scores of the subgroups derived from the interaction of the predictor variables (failing, repeating, and interpersonal values), we carried out a two-factor ANOVA with interaction.

## Results

### Delinquent behaviors and interpersonal values as a function of failing and repeating

Observing the mean scores for delinquent behavior and the different interpersonal values as a function of the variable failing, students who had failed a subject presented significantly higher mean scores in delinquent behaviors, recognition, and leadership, with small effect sizes (*d* ≤ 0.50), except for delinquent behaviors, where the effect was medium (*d* = 0.58). On the other hand, significantly higher scores were only found in the value benevolence for students who had never failed and, again in this case, the effect of the variable failing was small (*d* = 0.30).

When addressing gender, we observed that the same results were repeated in the groups of males and females, except that for the females, no differences were found in the mean score of the value leadership (see Table 1).

**Table 1:** **Difference of means in delinquent behaviors and interpersonal values in students who had not and who had failed**.

	**Did not fail**			**Failed**			**Statistical significance**		
**Total**	***N***	***M***	***SD***	***N***	***M***	***SD***	***t*_860_**	***p***	***d***
SIV-S	155	17.81	4.34	715	17.30	4.42	1.31	0.19	0.12
SIV-C	153	15.27	5.45	709	14.76	5.27	1.09	0.27	0.1
SIV-R	155	10.17	4.10	712	12.08	4.66	−4.71	0.00	0.42
SIV-I	153	17.95	5.68	711	17.21	5.79	1.45	0.14	0.13
SIV-B	154	19.16	5.90	712	17.43	5.83	3.32	0.01	0.3
SIV-L	151	9.39	5.12	708	11.08	4.90	−3.82	0.00	0.34
Delinquent behavior	156	1.01	2.28	729	2.46	3.46	−6.53	0.00	0.58
**Male**	***N***	***M***	***SD***	***N***	***M***	***SD***	***t*_424_**	***p***	***d***
SIV-S	64	17.92	4.40	369	17.13	4.45	1.31	0.19	0.18
SIV-C	63	15.10	5.61	363	14.35	5.14	1.05	0.29	0.14
SIV-R	63	11.05	4.39	368	13.04	4.58	−3.20	0.01	0.44
SIV-I	64	17.09	5.98	366	17.31	5.73	−0.27	0.78	0.04
SIV-B	64	18.67	6.13	365	15.99	5.85	3.35	0.01	0.46
SIV-L	63	9.84	5.41	367	12.04	5.03	−3.17	0.01	0.43
Delinquent behavior	64	1.50	3.11	377	3.34	4.10	−4.16	0.00	0.56
**Female**	***N***	***M***	***SD***	***N***	***M***	***SD***	***t*_434_**	***p***	***d***
SIV-S	91	17.74	4.32	346	17.48	4.39	0.49	0.62	0.06
SIV-C	90	15.40	5.36	346	15.19	5.39	0.33	0.74	0.04
SIV-R	92	9.58	3.80	344	11.06	4.53	−3.19	0.01	0.37
SIV-I	89	18.57	5.41	345	17.10	5.85	2.14	0.03	0.26
SIV-B	90	19.50	5.73	347	18.95	5.41	0.85	0.39	0.1
SIV-L	88	9.07	4.91	341	10.05	4.55	−1.77	0.07	0.21
Delinquent behavior	92	0.66	1.36	352	1.52	2.26	−4.60	0.00	0.54

SIV-S, Stimulus; SIV-C, Conformity; SIV-R, Acknowledgement; SIV-I, Independence; SIV-B, Benevolence; SIV-L, Leadership.

The analysis of the mean scores of interpersonal values and delinquent behaviors as a function of repeating/not repeating a course (see Table 2) revealed significantly higher scores in leadership and delinquent behaviors for repeaters, with a small effect for the variable repeating in both cases (*d* ≤ 0.50), whereas non-repeaters presented significantly higher scores in stimulation, also with a small effect for the variable repeating (*d* = 0.29). The same thing occurred in the analysis as a function of gender in the group of males and females, except that in the males, no differences in the mean scores of leadership were obtained.

**Table 2:** **Difference of means in delinquent behaviors values and interpersonal values in repeater and non-repeater students**.

	**Non-repeaters**			**Repeaters**			**Statistical significance**		
**Total**	***N***	***M***	***SD***	***N***	***M***	***SD***	***t*_860_**	***p***	***d***
SIV-S	600	17.79	4.42	270	16.51	4.27	3.99	0.00	0.29
SIV-C	594	14.85	5.39	268	14.86	5.12	−0.03	0.98	0
SIV-R	599	11.66	4.60	268	11.91	4.66	−0.75	0.45	0.05
SIV-I	596	17.29	5.79	268	17.45	5.75	−0.37	0.71	0.03
SIV-B	600	17.85	5.94	266	17.48	5.74	0.86	0.39	0.06
SIV-L	592	10.35	5.10	267	11.75	4.56	−3.84	0.00	0.28
Delinquent behavior	612	1.82	2.96	273	3.06	3.91	−4.65	0.00	0.38
**Male**	***N***	***M***	***SD***	***N***	***M***	***SD***	***t*_424_**	***p***	***d***
SIV-S	296	17.68	4.44	137	16.32	4.33	2.98	0.01	0.31
SIV-C	291	14.32	5.39	135	14.76	4.80	−0.82	0.41	0.08
SIV-R	295	12.66	4.72	136	12.93	4.33	−0.56	0.57	0.06
SIV-I	296	17.31	5.89	134	17.19	5.50	0.21	0.83	0.02
SIV-B	295	16.40	6.14	134	16.38	5.60	0.03	0.97	0
SIV-L	294	11.42	5.33	136	12.36	4.64	−1.77	0.07	0.18
Delinquent behavior	302	2.56	3.69	139	4.18	4.47	−3.73	0.00	0.41
**Female**	***N***	***M***	***SD***	***N***	***M***	***SD***	***t*_434_**	***p***	***d***
SIV-S	304	17.90	4.40	133	16.71	4.22	2.64	0.01	0.27
SIV-C	303	15.36	5.35	133	14.95	5.45	0.72	0.47	0.08
SIV-R	304	10.69	4.26	132	10.87	4.78	−0.39	0.69	0.04
SIV-I	300	17.27	5.69	134	17.71	6.00	−0.73	0.46	0.08
SIV-B	305	19.26	5.38	132	18.60	5.69	1.16	0.24	0.12
SIV-L	298	9.29	4.64	131	11.11	4.41	−3.81	0.00	0.4
Delinquent behavior	310	1.11	1.73	134	1.89	2.80	−2.99	0.01	0.37

SIV-S, Stimulus; SIV-C, Conformity; SIV-R, Acknowledgement; SIV-I, Independence; SIV-B, Benevolence; SIV-L, Leadership.

### Delinquent behaviors in students with high and low scores in interpersonal values

Table 3 presents the differences in the presence of delinquent behaviors between students with high and low scores in the diverse SIV scales, in the total sample, as well as according to gender, failing, and repeating a course. For the total sample, all the scales presented significant differences except for the value stimulation. Thus, students with high levels of recognition, independence, and leadership showed significantly higher levels of delinquent behaviors, with a small effect size of the values recognition and leadership (*d* = 0.28 and *d* = 0.49, respectively), whereas the effect size of the value independence was medium (*d* = 0.53). Students with low levels of conformity and benevolence displayed significantly higher levels of delinquent behaviors, in both cases with a medium effect size of both values (*d* ≥ 0.50).

**Table 3:** **Difference of means in delinquent behaviors in students low and high levels in interpersonal values**.

**SIV**		**Low Level**			**High Level**			**Statistical significance**		
**Total**		***N***	***M***	***SD***	***N***	***M***	***SD***	***t*_522_**	***p***	***d***
Delinquent behavior	SIV-S	233	2.18	3.55	291	1.91	2.58	0.97	0.33	0.09
	SIV-C	235	3.32	3.76	227	1.37	2.57	6.55	0.00	0.61
	SIV-R	218	1.82	2.90	238	2.78	3.81	−3.02	0.01	0.28
	SIV-I	237	1.33	2.52	268	3.11	3.94	−6.11	0.00	0.53
	SIV-B	262	3.14	3.89	248	1.16	1.98	7.30	0.00	0.64
	SIV-L	240	1.53	2.58	246	3.15	3.87	−5.41	0.00	0.49
**Male**		***N***	***M***	***SD***	***N***	***M***	***SD***	***t*_251_**	***p***	***d***
Delinquent behavior	SIV-S	115	3.23	4.31	138	2.29	3.03	1.96	0.05	0.26
	SIV-C	127	4.32	4.32	105	2.18	3.32	4.16	0.00	0.55
	SIV-R	82	2.51	3.33	151	3.42	4.31	−1.79	0.07	0.23
	SIV-I	111	2.07	3.32	133	4.02	4.62	−3.82	0.00	0.48
	SIV-B	167	3.61	4.25	91	1.70	2.61	4.45	0.00	0.51
	SIV-L	94	2.37	3.43	161	3.84	4.31	−2.98	0.01	0.37
**Female**		***N***	***M***	***SD***	***N***	***M***	***SD***	***t*_269_**	***p***	***d***
Delinquent behavior	SIV-S	118	1.15	2.19	153	1.56	2.05	−1.58	0.11	0.19
	SIV-C	108	2.15	2.53	122	0.66	1.35	5.47	0.00	0.75
	SIV-R	136	1.40	2.53	87	1.66	2.38	−0.74	0.46	0.11
	SIV-I	126	0.68	1.17	135	2.22	2.89	−5.71	0.00	0.69
	SIV-B	95	2.31	2.98	157	0.84	1.41	4.49	0.00	0.69
	SIV-L	146	0.99	1.63	85	1.84	2.38	−2.89	0.01	0.44
**Did not fail**		***N***	***M***	***SD***	***N***	***M***	***SD***	***t*_96_**	***p***	***d***
Delinquent behavior	SIV-S	38	1.29	3.82	60	0.84	1.21	0.86	0.39	0.18
	SIV-C	40	1.50	1.98	44	0.55	0.99	2.75	0.01	0.61
	SIV-R	57	0.88	1.46	22	0.78	1.02	0.29	0.77	0.07
	SIV-I	35	0.52	0.85	52	1.67	3.21	−2.47	0.02	0.55
	SIV-B	36	0.89	1.72	64	0.64	1.20	0.85	0.39	0.18
	SIV-L	58	0.64	1.22	24	1.25	2.05	−1.36	0.18	0.41
**Failed**		***N***	***M***	***SD***	***N***	***M***	***SD***	***t*_424_**	***p***	***d***
Delinquent behavior	SIV-S	195	2.35	3.48	231	2.19	2.77	0.54	0.59	0.05
	SIV-C	195	3.69	3.93	183	1.56	2.79	6.12	0.00	0.63
	SIV-R	161	2.16	3.20	216	2.98	3.93	−2.23	0.03	0.23
	SIV-I	202	1.48	2.68	216	3.46	4.03	−5.96	0.00	0.58
	SIV-B	226	3.49	4.02	184	1.34	2.16	6.94	0.00	0.69
	SIV-L	182	1.82	2.82	222	3.35	3.97	−4.52	0.00	0.45
**Non-repeater**		***N***	***M***	***SD***	***N***	***M***	***SD***	***t*_361_**	***p***	***d***
Delinquent behavior	SIV-S	142	1.64	2.99	221	1.78	2.50	−0.49	0.62	0.05
	SIV-C	165	3.25	3.74	161	.96	1.69	7.15	0.00	0.79
	SIV-R	156	1.38	2.42	156	2.61	3.68	−3.50	0.01	0.4
	SIV-I	170	1.17	2.18	183	2.61	3.43	−4.75	0.00	0.5
	SIV-B	172	2.73	3.55	178	0.98	1.76	5.80	0.00	0.63
	SIV-L	191	1.23	2.07	145	2.83	3.78	−4.60	0.00	0.55
**Repeater**		***N***	***M***	***SD***	***N***	***M***	***SD***	***t*_159_**	***p***	***d***
Delinquent behavior	SIV-S	91	3.01	4.16	70	2.30	2.82	1.29	0.19	0.2
	SIV-C	70	3.50	3.82	66	2.36	3.81	1.74	0.08	0.3
	SIV-R	62	2.94	3.65	82	3.09	4.06	−0.23	0.81	0.04
	SIV-I	67	1.75	3.20	85	4.19	4.71	−3.80	0.00	0.6
	SIV-B	90	3.91	4.37	70	1.60	2.41	4.25	0.00	0.64
	SIV-L	49	2.72	3.79	101	3.60	3.98	−1.30	0.19	0.23

SIV-S, Stimulus; SIV-C, Conformity; SIV-R, Acknowledgement; SIV-I, Independence; SIV-B, Benevolence; SIV-L, Leadership.

In the analysis as a function of gender, we observed that males and females with high levels of independence and leadership both presented significantly higher mean levels of delinquent behaviors, with a small effect size in all cases (*d* ≤ 0.50), except for the females in the value independence, where the effect of delinquent behaviors was medium (*d* = 0.69).

Regarding the variable failing, as in the total sample, the students who had failed and who presented high levels in recognition, independence, and leadership also displayed significantly levels higher in antisocial behaviors with a small effect size (*d* ≤ 0.50), except for the value independence, where the effect was medium (*d* = 0.58). In the group of students who did not fail, only the value independence had an effect on delinquent behaviors, with a medium effect (*d* = 0.58); the mean level of delinquent behaviors was statistically higher among students with high levels of independence. In the group of students who had failed, those with low levels of conformity and benevolence obtained significantly higher mean scores in antisocial behaviors, with a medium effect size for both values (*d* ≥ 0.50). This same result was observed in the group of students who did not fail, but only for the value conformity, with a medium effect of this value on delinquent behavior.

Lastly, with regard to repeating a course, non-repeaters who scored high on the scales of recognition, independence, and leadership also obtained significantly higher mean levels of delinquent behaviors, with effect sizes of *d* = 0.40, *d* = 0.50, and *d* = 0.55, respectively. This effect was also observed for the value independence among repeaters. Likewise, non-repeaters with low levels of conformity and benevolence presented higher mean levels of delinquent behaviors, with a medium effect size for both values (*d* ≥ 0.50). This same relation was observed among repeaters between the value benevolence and delinquent behaviors, with the same effect size as in the non-repeaters.

### Do interpersonal values predict delinquent behaviors?

Table 4 presents the probability of presenting high levels of delinquent behavior derived from the binary logistic regression in the total sample, considering the variables gender, failing, and repeating. The correct percentages of classification ranged between 57.4 and 69.8% for recognition and conformity, respectively. Regarding gender, for males, the correct classification ranged from 57.7% for the factor leadership to 67.4% for the factor conformity. With regard to the females, the correct classification ranged between 71.6% for independence and 74.4% for conformity. Concerning failing, the percentages for those who had failed a subject ranged from 62.2% for leadership to 70.6% for conformity, but recognition did not enter the model. In the group of students who had never failed, the correct percentages ranged from 80.3% for independence to 81.4% for conformity. In the analysis of the groups of repeaters and non-repeaters, the levels of correct classification ranged between 64% for recognition and 72.1% for conformity. In the group of repeaters, the correct classification ranged from 72.6% for the factor independence to 69.6% for the factor benevolence. Nagelkerke's *R*^2^ ranged between 0.02 for the females in the factor leadership and 0.28 for the non-repeaters in the factor conformity.

**Table 4:** **Logistic regression of the probability of high delinquent behaviors**.

**Total**	***B***	***SE***	**Wald**	***p***	**OR**	**95% CI**	**Nagelkerke *R*^2^**	**Correctly classified %**
SIV-C (low level)	−1.73	.25	49.393	0.00	0.18	0.11–0.29	0.21	69.8
SIV-R (high level)	0.57	0.22	6.497	0.01	1.77	1.14–2.76	0.03	57.4
SIV-I (high level)	1.52	0.24	40.718	0.00	4.58	2.87–7.30	0.16	66.7
SIV-B (low level)	−1.58	0.24	43.749	0.00	0.21	0.13–0.33	0.17	67.1
SIV-L (high level)	1.21	0.23	27.461	0.00	3.34	2.13–5.24	0.11	64
**Male**	***B***	***SE***	**Wald**	***p***	**OR**	**IC 95%**	**Nagelkerke *R*^2^**	**Correctly classified %**
SIV-C (low level)	−1.41	0.33	18.34	0.00	0.24	0.13–0.47	0.14	67.4
SIV-I (high level)	1.14	0.32	12.92	0.00	3.14	0.00–3.14	0.10	64
SIV-B (low level)	−1.02	0.32	9.96	0.01	0.36	0.19–0.68	0.07	61.9
SIV-L (high level)	0.08	0.02	13.39	0.00	1.09	1.04–1.14	0.06	57.7
**Female**	***B***	***SE***	**Wald**	***p***	**OR**	**IC 95%**	**Nagelkerke *R*^2^**	**Correctly classified %**
SIV-C (low level)	−2.22	0.44	25.12	0.00	0.11	0.04–0.26	0.27	74.4
SIV-I (high level)	2.01	0.40	25.23	0.00	7.45	3.40–16.32	0.23	71.6
SIV-B (low level)	−1.85	0.38	23.14	0.00	0.16	0.07–0.33	0.20	73.7
SIV-L (high level)	0.06	0.03	4.20	0.06	0.03	1.00–1.12	0.02	74
**Did not fail**	***B***	***SE***	**Wald**	***p***	**OR**	**IC 95%**	**Nagelkerke *R*^2^**	**Correctly classified %**
SIV-C (low level)	−1.58	0.74	4.55	0.03	0.21	0.05–0.88	0.13	81.4
SIV-I (constant)	−1.41	0.32	19.08	0.00	0.24	–	–	80.3
**Failed**	***B***	***SE***	**Wald**	***p***	**OR**	**IC 95%**	**Nagelkerke *R*^2^**	**Correctly classified %**
SIV-C (low level)	−1.77	0.27	43.33	0.00	0.17	0.10–0.29	0.22	70.6
SIV-I (high level)	1.67	0.26	41.74	0.00	5.33	3.21–8.85	0.19	69.3
SIV-B (low level)	−1.63	0.26	37.57	0.00	0.20	0.12–0.33	0.18	68.3
SIV-L (high level)	1.04	0.25	17.30	0.00	2.82	1.73–4.59	0.08	62.2
**Non-repeater**	***B***	***SE***	**Wald**	***p***	**OR**	**IC 95%**	**Nagelkerke *R*^2^**	**Correctly classified %**
SIV-C (low level)	−2.16	0.33	43.43	0.00	0.11	0.06–0.22	0.28	72.1
SIV-R (high level)	1.01	0.29	12.01	0.01	2.76	1.55–4.89	0.07	64
SIV-I (high level)	1.41	0.29	22.68	0.00	4.08	2.29–7.27	0.13	65.3
SIV-B (low level)	−1.58	0.30	27.22	0.00	0.20	0.11–0.37	0.16	66.4
SIV-L (high level)	1.18	0.29	16.64	0.00	3.25	1.84–5.73	0.10	66.5
**Repeater**	***B***	***SE***	**Wald**	***p***	**OR**	**IC 95%**	**Nagelkerke *R*^2^**	**Correctly classified %**
SIV-I (high level)	1.95	0.44	19.88	0.00	7.00	2.98–16.47	0.25	72.6
SIV-B (low level)	−1.63	0.41	15.56	0.00	0.20	0.09–0.44	0.19	69.6

SIV-S, Stimulus; SIV-C, Conformity; SIV-R, Acknowledgement; SIV-I, Independence; SIV-B, Benevolence; SIV-L, Leadership; B, coefficient; CI, 95% confidence interval.

The analysis and interpretation of the OR data obtained in total sample indicate that the probability of presenting high levels of delinquent behavior is: (a) 4.58 times higher in students with high independence, (b) 3.34 times higher in students with high leadership, (c) 1.77 times higher in students with high recognition, (d) 0.21 times lower in students with high benevolence, and (e) 0.18 times lower in students with high conformity.

In the analysis as a function of gender and the variables failing and repeating, the probability of presenting high levels of delinquent behavior is: (a) 0.24 (males), 0.11 (females), 0.21 (NOT failing), 0.17 (Failing), and 0.11 (Non-repeater) times lower in students with high conformity; (b) 0.07 (Non-repeater) times higher in students with high recognition; (c) 3.14 (males), 7.45 (females), 0.24 (NOT failing), 5.33 (Failing), 4.08 (Non-repeater), and 7 (Repeater) times higher in students with high independence; (d) 0.36 (males), 0.16 (females), 0.28 (Failing), 0.20 (Non-repeater), and 0.20 (Repeater) times lower in students with high benevolence; and (e) 1.09 (males), 0.03 (females), 2.82 (Failing), and 3.25 (Non-repeater) times higher in students with high leadership.

### Interpersonal values predicting delinquent behaviors: with and without interaction with the variables failing and repeating

**Figure 1:**
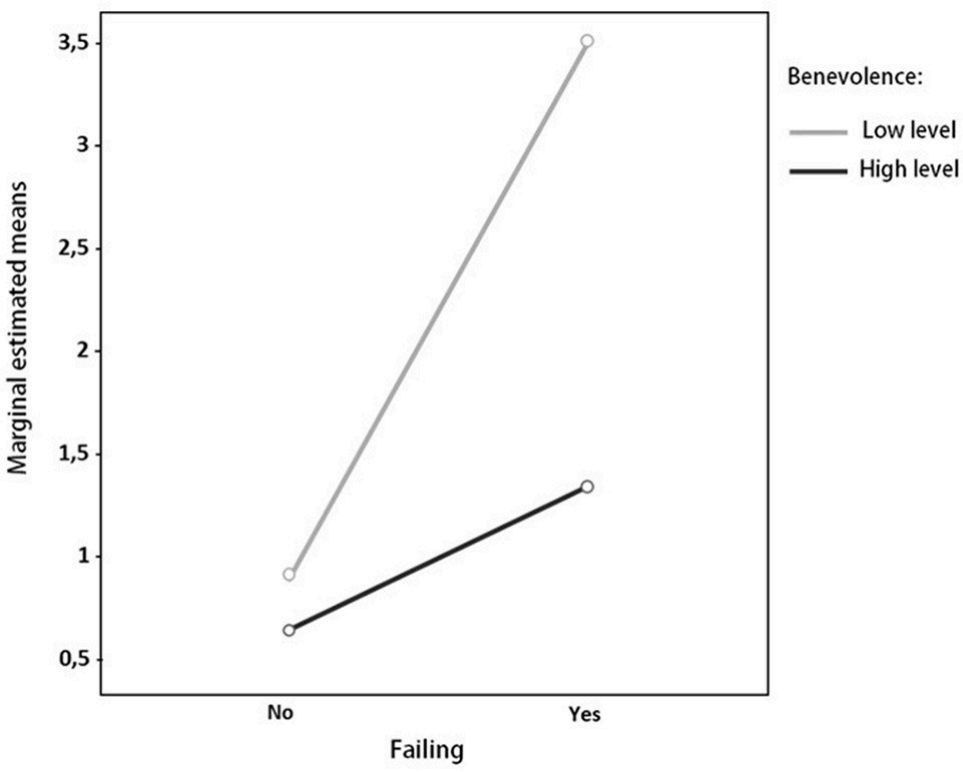
**Two-factor ANOVA with interaction: Failing and interpersonal value benevolence**.

We analyzed the scores obtained in the five factors as a function of both variables (failing and repeating), by means of a two-factor ANOVA with interaction, obtaining a significant interaction only between the factor benevolence and the variable failing, *F*_(1, 509)_ = 7.4, *p* = 0.01, *R*^2^ = 0.13, as shown in Figure 1.

## Discussion

With regard to the first goal of this study, we note that the students who failed and the repeaters present higher means of delinquent behaviors, both in males and in females, with a medium effect of the variable failing (*d* ≤ 0.79), and a small effect for the variable repeating (*d* ≤ 0.50), respectively. These results are in line with those found by other studies relating low academic performance with delinquent actions (Briggs-Gowan and Carter, 2008; Gázquez and Pérez-Fuentes, 2010; Preddy and Fite, 2012).

Not only students who fail, but also the repeaters present higher means in the values of recognition, as well as in the value of leadership. These differences are maintained in the groups of males and females who fail a subject, but only for the value recognition, with a small effect size (*d* ≤ 0.50). On the other hand, students who had not failed obtained higher scores in the value benevolence, and non-repeaters in the value stimulation, with small effects (*d* ≤ 0.50) of both variables, failing and repeating, respectively. Whereas in other studies, the value leadership is understood as prosocial leadership and is related to competence and social adaptation in adolescents (Jiménez and López-Zafra, 2011), in our study, leadership has a negative interpretation in the questionnaire that assesses it, because it refers to exerting authority over other people, that is, a position of control or power. Therefore, it may be appropriate to use another type of instrument that would allow us to measure this value positively in order to analyze the influence of prosocial leadership on adolescents' delinquent behavior.

The results achieved for our second goal reveal that students with high levels of recognition, independence, and leadership show significantly higher levels of delinquent behaviors, regardless of the variables failing and repeating and of gender concerning independence and leadership. Moreover, students with low levels of conformity and benevolence obtained significantly higher levels of delinquent behaviors, regardless of the variables failing and repeating. That is, individuals who like to be acknowledged, admired and approved of by others; who use their own criteria to decide what they have a right to do; who exert authority and power over others; who do not follow socially correct or appropriate norms or rules; and who are not very generous and do not help others-all these individuals present higher levels of delinquent behaviors.

Lastly, with regard to the third goal, the probability of presenting a high level of delinquent behavior is greater among students with: high independence, high leadership, high recognition, low benevolence, and low conformity. These five negative predictors should be the target of intervention in order to eliminate delinquent behavior.

Ultimately, we note the great importance of the interaction of benevolence and failing, which, when levels of benevolence are low and the student has failed some subject, leads to a considerable increase in delinquent behavior.

A limitation of this study is the sample, which, although representative, only included students from secondary education. A possible goal of future research is to carry out this same study with higher educational levels or in non-regulated studies, to determine whether the influence of interpersonal values on delinquent behaviors changes or remains the same.

Therefore, although the present study presents some limitations to be taken into account in future studies, it can be considered a precursor in a new line of research to clarify the relation between delinquent behavior and violence, adding to the diverse studies that have not clarified the relation between them. It may also be of great interest to the educational community because it contributes relevant data for the design of interventions promoting protector factors and reducing risk factors, for example, in the peer group (Knecht et al., 2010; Paciello et al., 2013). It is also of interest to parents and in order to elaborate programs targeting the parents, because, as indicated, family factors are highly involved in the origin of adolescents' delinquent behaviors (Martínez et al., 2013; Tolan et al., 2013).

## Author contributions

The authors incorporated worked with AMM and ABBM in the literature search. The distribution of tasks would be as follows: MMJ and MCPF (Drafting and analysis of data). AMM, ABBM, and MMSM (bibliographic search). ALR Helped in the realization of the changes requested by the reviewer).

## Funding

This work is the result of Research Project P08-SEJ-04305, co-financed by the Consejería de Innovación, Ciencia y Empresa (Council of Innovation, Science and Enterprise) of the Junta of Andalucía and FEDER.

### Conflict of interest statement

The authors declare that the research was conducted in the absence of any commercial or financial relationships that could be construed as a potential conflict of interest. The reviewer DÁ and the handling Editor declared their shared affiliation, and the handling Editor states that the process nevertheless met the standards of a fair and objective review.
